# In-vitro biological evaluation of 3,3′,5,5′-tetramethoxy-biphenyl-4,4′-diol and molecular docking studies on trypanothione reductase and Gp63 from *Leishmania amazonensis* demonstrated anti-leishmania potential

**DOI:** 10.1038/s41598-023-34124-9

**Published:** 2023-04-28

**Authors:** Jéseka G. Schirmann, Bruna T. S. Bortoleti, Manoela D. Gonçalves, Fernanda Tomiotto-Pellissier, Priscila G. Camargo, Milena M. Miranda-Sapla, Camilo H. S. Lima, Marcelle L. F. Bispo, Idessania N. Costa, Ivete Conchon-Costa, Wander R. Pavanelli, Robert F. H. Dekker, Aneli M. Barbosa-Dekker

**Affiliations:** 1 Departamento de Química, Centro de Ciências Exatas, Universidade Estadual de Londrina, Londrina, PR Brazil; 2 Fiocruz, Programa de Pós-Graduação em Biociências e Biotecnologia, Instituto Carlos Chagas, Curitiba, PR Brazil; 3 Departamento de Ciências Patológicas, Centro de Ciências Biológicas, Universidade Estadual de Londrina, Londrina, PR Brazil; 4 Instituto de Química, Universidade Federal Do Rio de Janeiro, Rio de Janeiro, RJ Brazil; 5 Programa de Pós-Graduação em Engenharia Ambiental, Universidade Tecnológica Federal do Paraná, Câmpus de Londrina, Londrina, PR Brazil

**Keywords:** Chemical biology, Computational biology and bioinformatics, Drug discovery, Microbiology, Diseases

## Abstract

Available treatments for leishmaniasis have been widely used since the 1940s but come at a high cost, variable efficacy, high toxicity, and adverse side-effects. 3,3′,5,5′-Tetramethoxy-biphenyl-4,4′-diol (TMBP) was synthesized through laccase-catalysis of 2,6-dimethoxyphenol and displayed antioxidant and anticancer activity, and is considered a potential drug candidate. Thus, this study aimed to evaluate the anti-leishmanial effect of TMBP against promastigote and amastigote forms of *Leishmania (L.) amazonensis* and investigated the mechanisms involved in parasite death. TMBP treatment inhibited the proliferation (IC_50_ 0.62–0.86 µM) and induced the death of promastigote forms by generating reactive oxygen species and mitochondrial dysfunction. In intracellular amastigotes, TMBP reduced the percentage of infected macrophages, being 62.7 times more selective to the parasite (CC_50_ 53.93 µM). TMBP did not hemolyze sheep erythrocytes; indicative of low cytotoxicity. Additionally, molecular docking analysis on two enzyme targets of *L. amazonensis*: trypanothione reductase (TR) and leishmanolysin (Gp63), suggested that the hydroxyl group could be a pharmacophoric group due to its binding affinity by hydrogen bonds with residues at the active site of both enzymes. TMBP was more selective to the Gp63 target than TR. This is the first report that TMBP is a promising compound to act as an anti-leishmanial agent.

## Introduction

Leishmaniasis is an infectious neglected tropical disease caused by species of protozoa belonging to the *Trypanosomatidae* family and the genus *Leishmania*, and affects about 700,000 to 1 million people in 98 countries^1–3^. Leishmaniasis can manifest in different clinical forms, including cutaneous and mucocutaneous ulcers that affect the skin and mucous membranes, respectively, and the visceral form, which affects the internal organs^4^.

Pentavalent antimonials such as meglumine antimonate (Glucantime) and sodium stibogluconate (Pentostam) are the first-choice drugs used to treat leishmaniasis. Second-line drugs, such as Amphotericin B (AmB), Paromomycin, Pentamidine, and Miltefosine, are therapeutic alternative in cases of parasite resistance or contraindication to antimonials^5,6^. Although they have been widely used, the available drugs bear high costs, variable efficacies, high toxicity, and serious adverse effects such as fatigue, increased serum liver enzyme levels, pancreatitis, myalgia, pancytopenia, arthralgia, gastrointestinal dysfunction, diffuse muscle pain, stiffening of the joints, cardiac arrhythmias, reversible renal failure, and cardiotoxicity^6,7^. In this respect, developing new drugs against *Leishmania* with good efficacy and a safety profile is still needed.

Computational tools such as molecular docking are used to identify the possible molecular mechanisms of action of bioactive substances^8–10^. Several studies highlighted the concept of multiple targets for *Leishmania* diseases, as the trypanosomatid family shares many common characteristics, such as gene conservation and high amino acid identity among proteins^11–13^. In this context, two essential and attractive targets from *Leishmania* spp*.* are trypanothione reductase (TR) and leishmanolysin, which are involved in the defense against oxidants and the transport and survival of the parasite, respectively.

TR (EC 1.8.1.12) has multiple functions for the parasite, such as the defense against oxidant agents and protein thiol-redox homeostasis. This enzyme promotes the activation of a cascade of events that neutralize generated reactive oxygen species (ROS) through catalytic reduction of trypanothione disulfide to trypanothione dithiol^9,14,15^. In addition, there were recent advancements in synthetic compounds as oral therapeutic leads targeting the *Leishmania* TR. Water-soluble ferrocenylquinoline derivative (CQFC) was.reported to mediate the specific inhibition of TR in vivo, with a high inhibitory potency value (*K*_*i*_) as low as 0.87 ± 0.09 μM^16^. Also, Gold(I) complexes containing quinoline functionalized *N*-heterocyclic carbenes (AdO Et and AdT Et) showed a potent inhibitory effect against TR with IC_50_ values ranging from 1.0 to 7.8 μM^17^.

Leishmanolysin, or glycoprotein 63 (Gp63) (EC 3.4.24.36), is a zinc metalloprotease and the main protein component of the promastigote surface. This enzyme maximizes promastigote binding to internalize macrophages, facilitating their entry into these cells, and enhancing phagocytosis and parasite survival, thus contributing to the infectivity of *Leishmania*^18–20^. All these factors consolidate TR and Gp63 as attractive targets in designing antileishmanial drugs.

Phenolic compounds have extensively been studied for their biological activities, including as antileishmanial agents^21^. In this respect, biphenyl compounds are an important intermediate in organic chemistry that constitutes the structural moiety of many compounds presenting various pharmacological activities such as anti-inflammatory, diuretic, anti-diabetic, and antimicrobial^22,23^. The biphenyl, 3,3′,5,5′-tetramethoxy-biphenyl-4,4′-diol (TMBP), is a synthetic compound produced by laccase catalysis of 2,6-dimethoxyphenol (2,6-DMP)^24^. TMBP has been described as an antioxidant with the potential to inhibit oxidative processes in soybean biodiesel^24^ and also exhibits a stabilizing influence on soybean biodiesel^25^. TMBP has also been reported to have anticancer activity through its action on increasing the production of reactive oxygen species (ROS), causing mitochondrial depolarization, inducing cell cycle arrest in the G2/M phase, and leading to the death by direct apoptosis on the lung cancer cell line A549^26^. The anti-proliferative and apoptosis-inducing effects of another biphenyl compound, viz., 2,4,3′,4′-tetramethoxybiphenyl, also reported apoptosis acting through a mitochondrial/caspase pathway on human gastric cancer MGC-803 cells^27^. Phenolic compounds furthermore have been reported in the literature as exhibiting antioxidant, antimicrobial, anti-carcinogenic, and anti-inflammatory activities^28^. For example, gallic acid was described for its efficacy in inhibiting promastigotes forms of *L. amazonensis* (IC_50_ 14.48 μg/mL)^29^ and also acted as an inhibitor of the enzyme arginase (IC_50_ = 2.2 µM) involved in the polyamine pathway, and is considered a target to control *Leishmania* infections^30^. These findings encouraged us to investigate the leishmanicidal potential of the biphenyl TMBP, which has not yet been reported in the literature.

**Figure 1:**
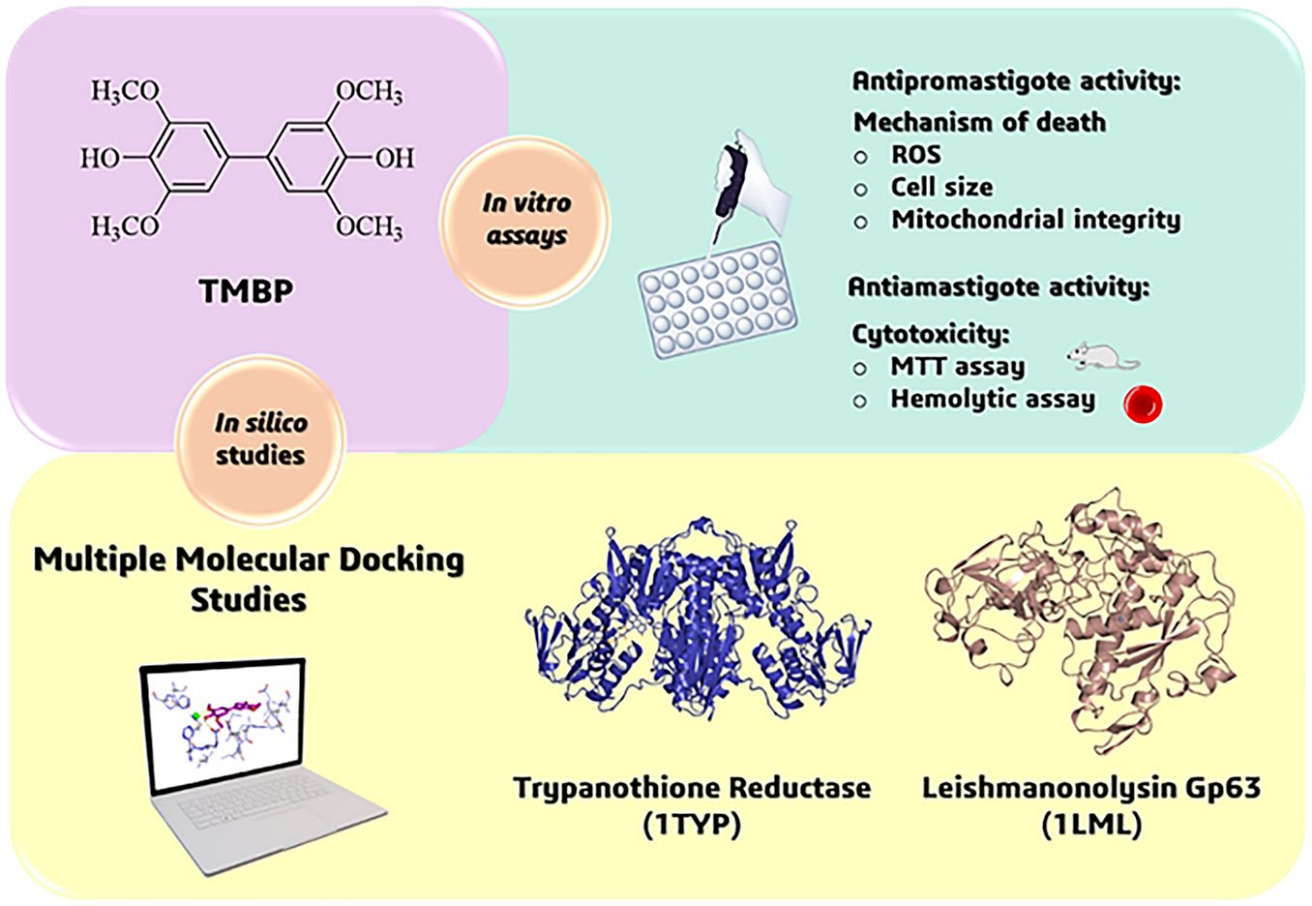
The experimental approach to leishmanicidal evaluation and molecular docking of TMBP.

Herein, we report for the first time on the biological activity of TMBP against the promastigote and amastigote forms of *L. amazonensis* using different experimental approaches. Furthermore, we investigate the possible mechanism of action of TMBP in the parasite's death, in addition to an investigation of its cytotoxicity on peritoneal macrophages. Finally, we performed consensual molecular docking of TMBP on TR and Gp63, two relevant targets in the parasite's life cycle (Fig. 1).

## Results and discussion

### TMBP exerts antileishmanial activity against *L. amazonensis* promastigote forms

**Figure 2:**
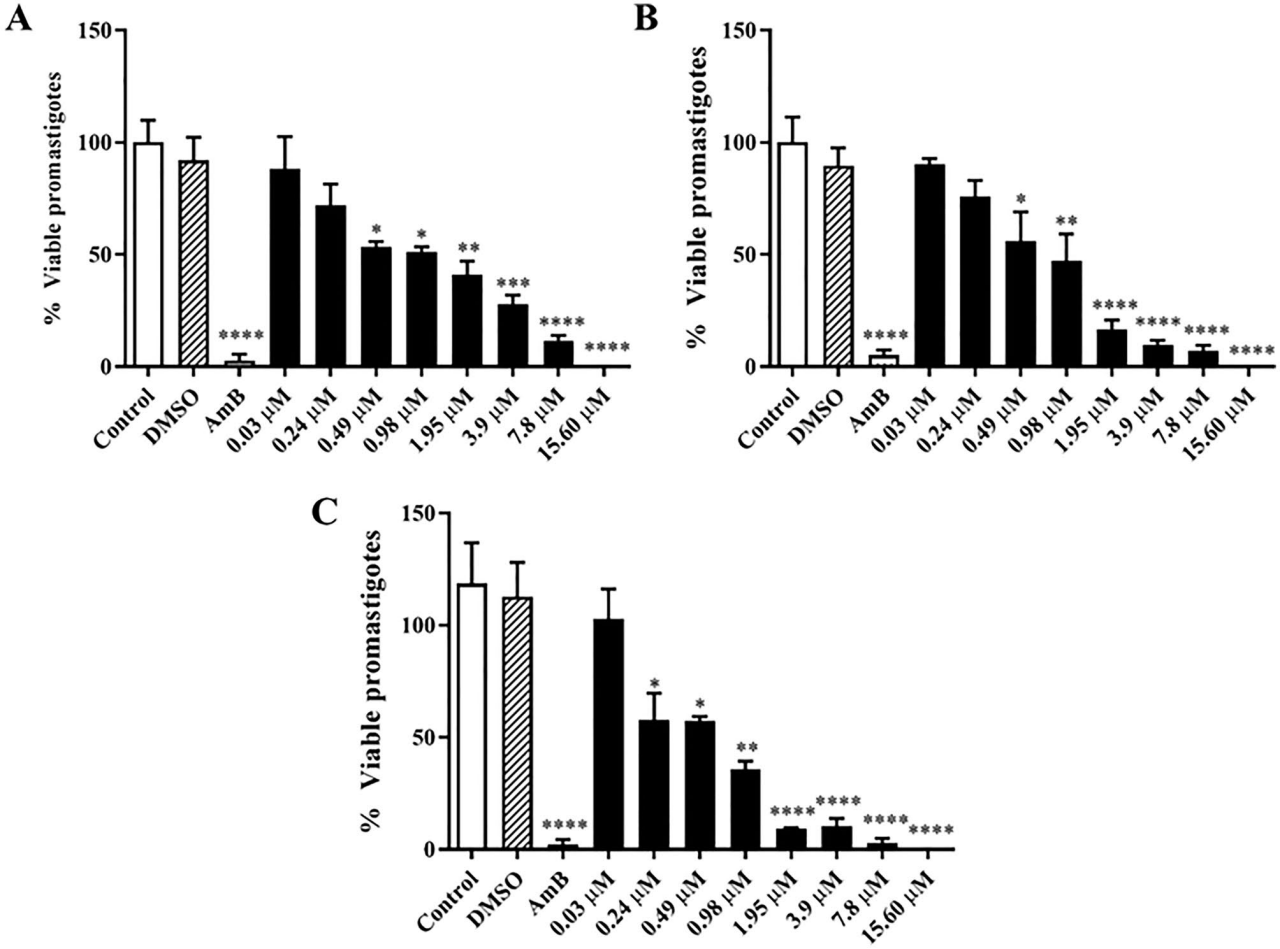
TMBP leishmanicidal activity (tested concentrations 0.03–15.6 μM) on promastigote forms of *L. amazonensis* at **(A)** 24 h, **(B)** 48 h, and **(C)** 72 h of treatment. Amphotericin B (AmB, 1 μM) was used as standard, and dimethyl sulfoxide (DMSO, 0.015%, v/v) as the compound vehicle. Data represent the mean ± SEM of three independent experiments performed in duplicate. *(*p* < 0.05); **(*p* ≤ 0.01); ***(*p* ≤ 0.001); and ****(*p* ≤ 0.0001) are statistical differences compared to control by Ordinary one-way ANOVA test.

The biphenyl TMBP used in this work was synthesized from 2,6-DMP in a laccase-catalyzed reaction, as previously reported by our research group. TMBP was characterized spectroscopically by ^1^H NMR, HRMS (ESI), and GC-MS^24^. The direct effect of TMBP on promastigote forms of *L. amazonensis* after 24, 48, and 72 h was measured in its presence or absence at different concentrations. After 24 and 48 h, TMBP significantly inhibited the viability of the parasites at concentrations ranging from 0.49 μM (*p* < 0.05, 24 and 48 h), 0.98 μM (*p* < 0.05, 24 h and *p* ≤ 0.01, 48 h), 1.95 μM (*p* ≤ 0.01, 24 h and *p* ≤ 0.0001, 48 h), 3.90 μM (*p* ≤ 0.001, 24 h and *p* ≤ 0.0001, 48 h), 7.80 μM (*p* ≤ 0.0001, 24 and 48 h) and 15.60 μM (*p* ≤ 0.0001, 24 and 48 h), with IC_50_ values of 0.86 ± 0.02 μM and 0.66 ± 0.06 μM, for 24 and 48 h, respectively (Fig. 2A,B). Treatment with DMBP for 72 h significantly inhibited the viability of the parasites at a lower concentration of 0.24 μM (*p* < 0.05), followed by 0.49 μM (*p* < 0.05), 0.98 μM (*p* ≤ 0.01), 1.95 μM (*p* ≤ 0.0001), 3.90 μM (*p* ≤ 0.0001), 7.80 μM (*p* ≤ 0.0001) and 15.60 μM (*p* ≤ 0.0001) with IC_50_ = 0.48 ± 0.06 μM (Fig. 2C). The IC_50_ values did not statistically differ between the times evaluated for the treatment by TMBP (data not shown).

In general, compounds with IC_50_ ≤ 10.00 µM are reported in the literature to have potential pharmacologic activity^31^. In this respect, TMBP showed potential antileishmanial activity by completely inhibiting the viability of the parasites, even in the shortest incubation time (24 h), and exhibiting low IC_50_ values, ranging from 0.48 to 0.86 μM.

**Figure 3:**
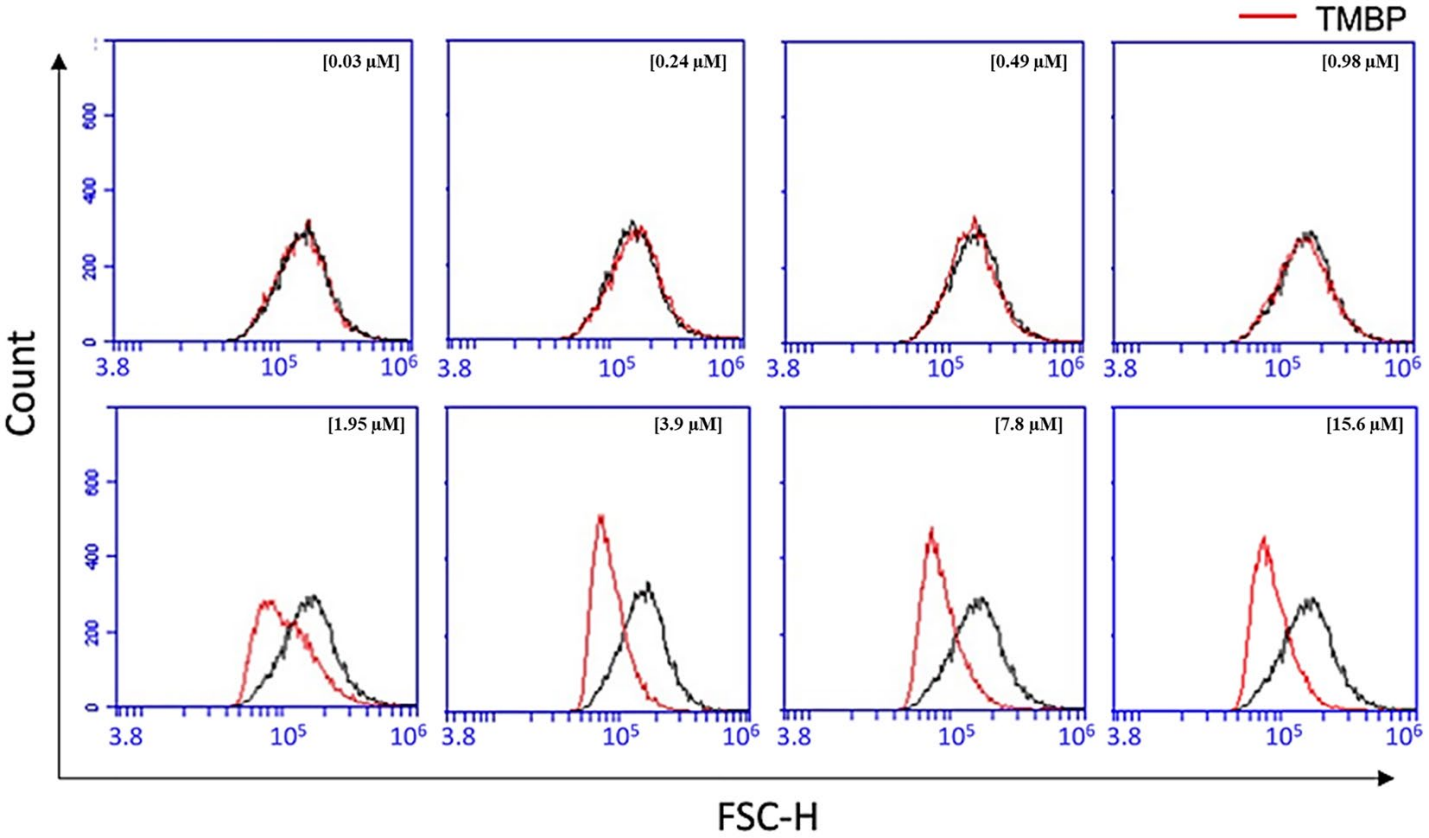
Cell size on promastigote forms from *L. amazonensis* treated with TMBP for 24 h. FSC-H was used to assess cell size at each of the concentrations of TMBP (0.03 to 15.6 µM). Black lines are the control group (non-treated parasites), and the red-line area corresponds to the treated group.

As cell shrinkage is a clear indication of cell death, primarily via apoptotic mechanisms^32–34^, we performed a flow cytometry analysis to confirm the antileishmanial effect of TMBP. The shortest period (24 h) was chosen for this set of experiments since there was no difference between the IC_50_ during the evaluated time. This analysis indicated a reduction in the cell size of the promastigotes treated with TMBP at concentrations ranging from 1.95 to 15.6 μM for 24 h (Fig. 3), suggesting the loss of cell volume, which is the predominant characteristic of apoptosis^33^. Apoptosis-related death has also been reported for other phenolic compounds, including gallic acid, quercetin, and rutin, through increasing nitric oxide production and causing apoptosis and DNA damage, which presented structural changes in cells that led to the death of *L. donovani*^35^.

On confirming the anti-promastigote effect of TMBP, we investigated the mechanisms of action involved in parasitic death by evaluating the production of reactive oxygen species (ROS) and the mitochondrial integrity in the parasites.

Due to their unique mitochondria, the *Leishmania* genus parasites require the maintenance of mitochondrial integrity for survival^36^. Studies have demonstrated that changes in mitochondrial integrity caused by drugs, such as antimony and sterol methenyl transferase (SMT) inhibitors, are related to cell death of *L. amazonensis*^37^.

**Figure 4:**
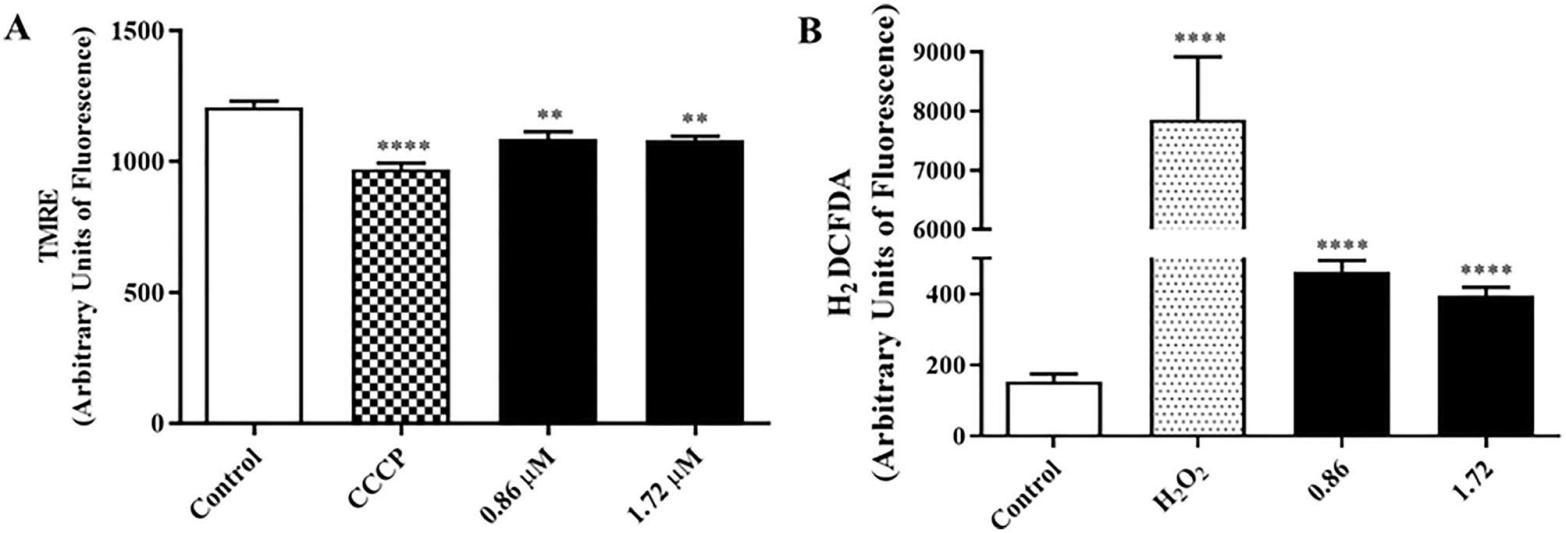
Analysis of the mechanism of death on promastigote forms from *L. amazonensis* by TMBP treatment (0.86 and 1.72 μM) in 24 h. **(A)** TMRE assay by fluorometric analysis of mitochondrial membrane potential. Control (non-treated parasites) and CCCP as a positive control. **(B)** ROS generation evaluation by H_2_DCFDA. Control (non-treated parasites) and H_2_O_2_ was the positive control. **(*p* ≤ 0.01) and ****(*p* ≤ 0.0001) are statistical differences compared to the control by Ordinary one-way ANOVA test.

The TMRE probe was used to assess the possible induction of alterations in the integrity of the mitochondrial potential of promastigote forms by TMBP. TMBP concentrations of 0.86 μM (IC_50_) and 1.72 μM (twice the IC_50_ value) were used in this and subsequent experiments. Treatment decreased the intensity of total fluorescence of TMRE (0.86 and 1.72 μM) compared to the control group, indicating loss of integrity of the mitochondria (Fig. 4A). This behavior was also observed for the depolarizing agent CCCP. These findings suggest that TMBP inhibits the viability of *L. amazonensis* promastigotes by affecting the parasite's mitochondrial functions.

We also evaluated the production of ROS in the promastigote forms treated with TMBP. The treated parasites demonstrated an increase in ROS levels compared to the control group at both analyzed TMBP concentrations; 0.86 and 1.72 μM (Fig. 4B). The same was observed for the positive control, H_2_O_2_. ROS can act directly on *Leishmania* spp., causing the oxidation of proteins, lipids, and nucleic acids^38^, thus causing damage to the mitochondria that leads to their depolarization^39^. These results demonstrated that the leishmanicidal effect of TMBP on the promastigote forms of *L. amazonensis* was associated with the production of ROS and mitochondrial dysfunction, causing the parasite's death.

### The effect of TMBP on the amastigote forms of *L. amazonensis*

**Figure 5:**
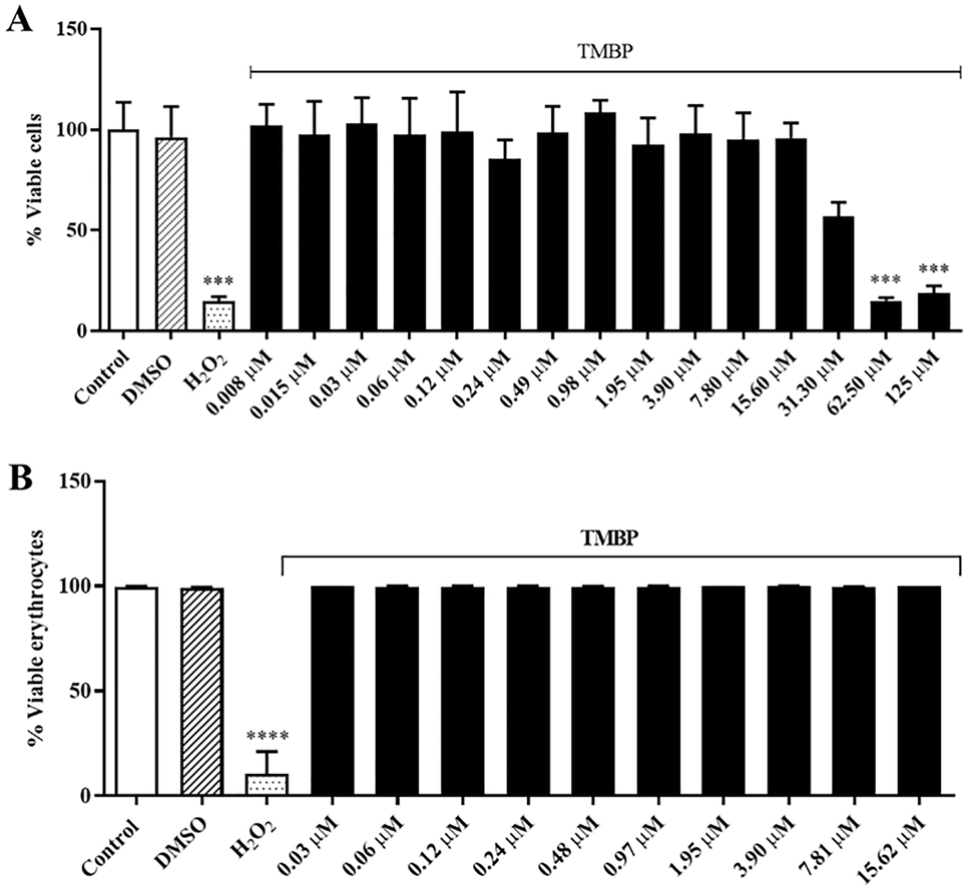
Cytotoxicity assays by TMBP treatment (0.03 to 125 μM). **(A)** Cell viability of peritoneal macrophages from BALB/c mice. **(B)** Cell viability of sheep erythrocytes. Control (non-treated parasites), H_2_O_2_ or H_2_O positive control, and DMSO as a compound diluent (0.015%). Data represents the mean ± SEM of three independent experiments performed in duplicate. ***(*p* ≤ 0.001) and ****(*p* ≤ 0.0001) are the statistical differences compared to the control by the Ordinary one-way ANOVA test.

To investigate the effect of TMBP on the amastigote forms of *L. amazonensis*, we first evaluated its cytotoxic action on two mammalian primary cells: murine macrophages and sheep erythrocytes. Concentrations of 0.03 to 15.60 µM did not alter the viability of peritoneal macrophages from BALB/c mice (Fig. 5A), presenting a CC_50_ value of 53.93 µM. The selectivity index of TMBP was determined and revealed that this compound was 62.7 times more selective for parasites than the host cells (BALB/c mice). The results of hemolytic activity (Fig. 5B), expressed as a percentage of viability, indicated that the tested concentrations of TMBP did not cause hemolysis of sheep erythrocytes. These observations agree with the findings reported by our group that TMBP did not cause lysis in sheep red blood cells and also did not result in the loss of viability of murine peritoneal macrophages, thereby reinforcing its potential use as a drug^26^.

**Figure 6:**
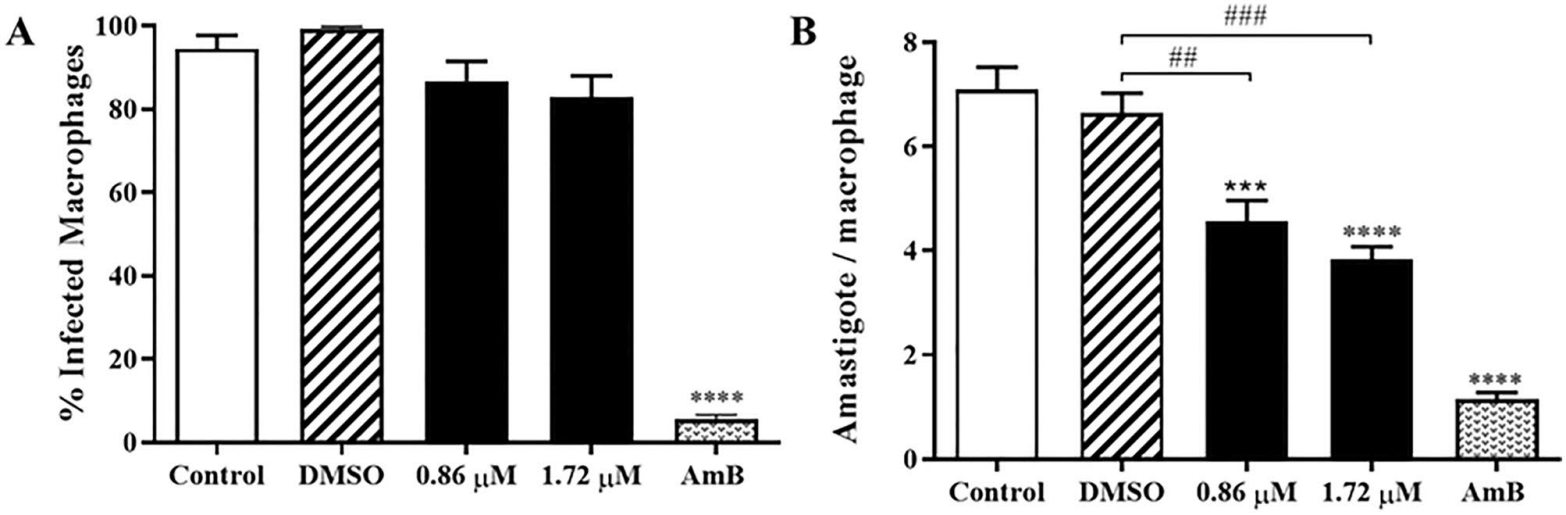
Infected macrophages by *L. amazonensis* treated with TMBP (0.86 µM and 1.72 µM). **(A)** Percentage of infected macrophages. **(B)** Evaluation of the numbers of amastigotes per macrophage. Negative control (RPMI 1640 medium), AmB as a positive control (1 µM), and 0.015% DMSO (diluent control). Values represent the mean ± SEM of three independent experiments performed in duplicate. Significant difference from the negative control ***(*p* ≤ 0.001); ****(*p* ≤ 0.0001). Significant difference from the ## diluent control (*p* ≤ 0.01) and ### (*p* ≤ 0.001).

Considering that TMBP reduced the proliferation of promastigote forms and was not toxic to macrophages, we decided to investigate its effect against the intracellular amastigote forms of *L. amazonensis*. Despite the results showing no statistically significant decrease in the percentage of infected cells (Fig. 6A), there was a reduction in the number of intracellular parasites per macrophage (Fig. 6B). Furthermore, the two TMBP concentrations evaluated significantly decreased the mean number of amastigotes per macrophage by 35.2 and 46.2% (*p* ≤ 0.001 and *p* ≤ 0.0001, respectively) compared to the control.

Macrophages are the primary host cells for *Leishmania* sp.^40^, and the intracellular localization of parasites represents an additional challenge for the treatment of this parasitosis since drugs must penetrate different cell layers, such as the cell membrane and the phagolysosome membrane to target the parasites specifically^41^. Thus, according to DNDi (Drugs for Neglected Diseases initiative), the ability to act on intracellular forms is required for potential antileishmanial drugs^42^.

In summary, TMBP acted on the intracellular parasite, reducing the number of viable amastigote forms in macrophages, allowing this compound to diffuse through the cell membrane, acting directly on the target without causing considerable toxic effects to the host cell. TMBP also did not demonstrate cytotoxic effects on peritoneal macrophages nor lysed sheep erythrocytes. Besides, cell death mechanisms resulted from the formation of reactive oxygen species and mitochondrial depolarization.

### Molecular docking studies

*Leishmania* species express abundant surface protein antigens in promastigotes. Gp63 promotes the parasite's interaction with the host's defensive systems in this context. Another relevant target is the enzyme TR, which is involved in thiol-redox homeostasis and acts in the parasite's defense against oxidative agents. Since our results demonstrated that TMBP could induce ROS production, decrease cell size, and cause damage to mitochondria, we decided to perform molecular docking studies to obtain insights into the putative effects of TMBP on these targets.

The consensus docking between at least two scoring functions that present the lowest value of RMSD (and < 2.0) for the identification of the pose and the intermolecular interactions on the binding site of LaGp63 were considered (Table 1) and presented a Fitness Score of 53.52.

**Table 1:** RMSD between poses results of the scoring functions applied in the GOLD program to LaGp3.

	Goldscore	ChemPLP	Chemscore	ASP
Goldscore	0	0.12	0.16	0.10
ChemPLP	0.12	0	0.23	**0.04**
Chemscore	0.16	0.23	0	0.21
ASP	0.10	**0.04**	0.21	0

Values highlighted in bold text are the best value of RMSD from the pose results between the score functions evaluated.

**Figure 7:**
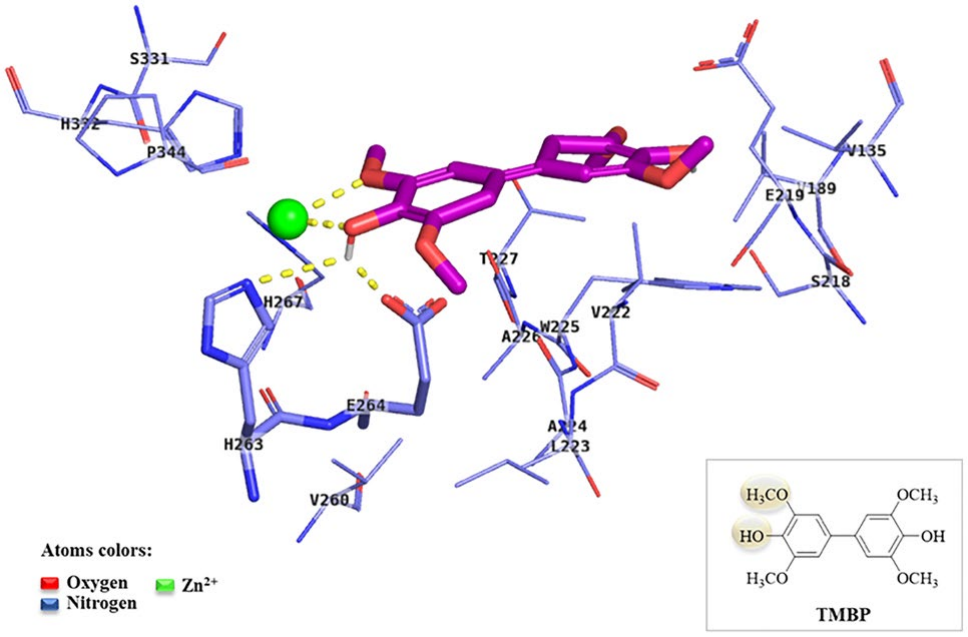
Interaction diagram of the consensual pose of TMBP (purple sticks and the inset for its chemical structure) at the active site of LaGp3. Blue sticks or lines represent interacting amino acid residues, and a green sphere represents the Zn^2+^ ion. The dashed yellow lines represent hydrogen bonding interactions with the amino acids or zinc.

The docking complex between the TMBP ligand and LaGp3 revealed that this biphenyl compound could interact with the enzyme by hydrophobic interactions involving amino acid residues: Val135, Val189, Ser218, Glu219, Val222, Leu223, Ala224, Trp225, Ala226, Val260, His267, Tyr327, Ser331, His332, and Pro344. Also, we observed hydrogen bonding at the active site, through hydroxyl groups of TBMP interacting with catalytic amino acids His263 and Glu264, in addition to metal interaction with the Zn^2+^ ion (Fig. 7). Furthermore, Glu264 is reported to assist the position of the catalytic residues His263, His267, and His332, allowing the coordination with a zinc ion. In this way, these observed interactions might cause the indirect inactivation of LaGp63.

**Figure 8:**
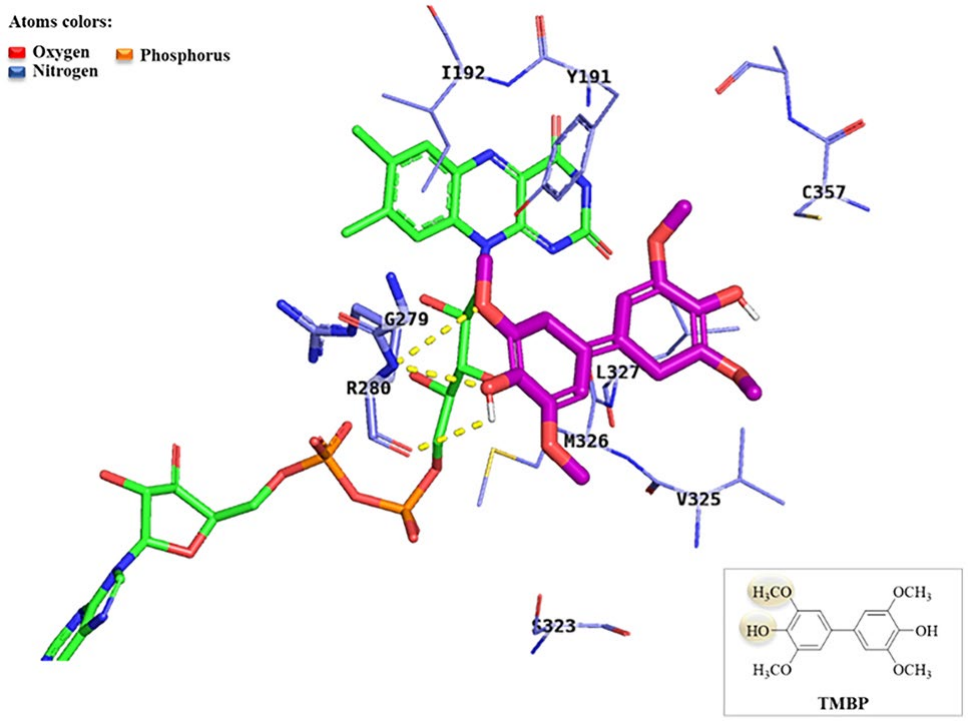
Interaction diagram of the consensual pose of TMBP (purple stick) in the active site of the enzyme LaTR. Blue sticks or lines represent interacting residues. A green stick represents the FAD cofactor. The dashed yellow lines represent the hydrogen bonding interactions with amino acids.

The clustering analysis of the docking simulation complexes between the ligand and LaTR showed that these compounds could interact with this enzyme by the hydrogen bonds present (Fitness Score = 46.22). TMBP accomplishes two relevant H-bond interactions with Arg280 and Gly279, in addition to hydrophobic interactions with Tyr191, Ile192, Val325, Ser323, Met326, Leu327, Cys357 (Fig. 8). In general, this possible affinity binding of TMBP at the active site and proximity to the enzyme's cofactor, FAD, might interfere with the TR enzyme's indirect inactivation of catalytic activity.

Combined analysis of both target enzymes (LaTR and LaGp63) suggests that the methoxyl and mainly the hydroxyl group of TMBP participated in all hydrogen bond interactions with crucial amino acid residues of both enzymes, with TMBP having a greater affinity for the LaGp63 target. In addition, the presence of the aromatic ring in the structure of TMBP increases its lipophilicity (cLogP = 2.44), facilitating the permeability of its entry into cell membranes of the promastigote or amastigote forms, where these enzymes can be found. Consequently, these results suggest that this biphenyl compound should be further studied for its antileishmanial activity.

## Materials and methods

### Production of TMBP

3,3′,5,5′-Tetramethoxy-biphenyl-4,4′-diol was previously reported to be synthesized from 2,6-dimethoxyphenol (Sigma-Aldrich, St Louis, MO, USA) in a reaction catalyzed by the laccase from *Botryosphaeria rhodina* MAMB-05, as described by Schirmann et al. (2018)^24^.

### Anti-leishmanial activity

#### *Leishmania (Leishmania) amazonensis* maintenance

Promastigote forms of *L. (L.) amazonensis* (MHOM/BR/1989/166MJO) were maintained in a culture medium comprising 199 (GIBCO, Invitrogen, New York, USA) pH 7.18–7.22 supplemented with 10% (w/v) fetal bovine serum (FBS) (GIBCO), 10 mM-HEPES buffer, 0.1% human urine, 0.1% L-glutamine, 10 U/mL penicillin, 10 μg/mL streptomycin (GIBCO), and 10% (w/v) sodium bicarbonate. The cell culture was maintained at 25 °C in a 25 cm^2^ culture flask. All experiments used promastigote forms at the stationary growth phase (5-day cultures).

#### Animals and ethics committee

The experiments using BALB/c mice were kindly provided by the Carlos Chagas Institute Fiocruz-PR, Curitiba, Brazil, and were approved by the Ethics Committee for Animal Experimentation of State University of Londrina (Protocol 4496.2018.81), according to the Brazilian federal law (11.794/2008, Decreto no 6.899/2009). Furthermore, the animals weighed approximately 25–30 g, and 6–8 weeks of age were kept under sterile conditions in a controlled and disinfected environment, with access to sterile water, food, and cage floor wood shavings, in compliance with the "Principles of Laboratory Animal Care" formulated by the National Society for Medical Research and the "Guide for the Care and Use of Laboratory Animals" prepared by the National Academy of Sciences, USA. The experiment involving animals follows the recommendations described in the ARRIVE guidelines.

#### Antipromastigote assay 

*L. amazonensis* promastigote forms (1 × 10^6^ cells/mL) were treated with TMBP at concentrations ranging from 0.03; 0.24; 0.49; 0.98; 1.95; 3.9; 7.8 to 15.6 µM. Parasites were counted on a Neubauer chamber after 24, 48, and 72 h of treatment. *L. amazonensis* promastigote maintained on medium 199 was used as the control, dimethyl sulfoxide (DMSO) 0.015% as a vehicle (v/v), and amphotericin B (AmB, 1 μM) as a positive control^43^. Three independent experiments were carried out in duplicate.

#### Determination of the cell size of the parasites

*L. amazonensis* promastigotes forms (10^6^ cells/mL) were treated with TMBP (0.03; 0.24; 0.49; 0.98; 1.95; 3.9; 7.8, and 15.6 µM) and incubated for 24 h at 24 °C. Afterward, the promastigotes were collected and washed with PBS, then analyzed by flow cytometry using a BD Accuri™ C6 Plus flow cytometer. The forward scatter-heights (FSC-H) represent the cell sizes. A total of 10,000 events were acquired in the region corresponding to the parasites^44^.

#### Determination of the IC_50_, CC_50,_ and Selectivity Index (SI)

The concentration of TMBP capable of inhibiting 50% of promastigote forms in culture (IC_50_) and the concentration responsible for causing the death of 50% of peritoneal macrophages (CC_50_) was calculated by non-linear regression using GraphPad software (Inc., USA, 5.00) from the data obtained in Sects. 2.9 and 2.14, respectively. In addition, the values corresponding to the IC_50_ and twice this concentration (2 × IC_50_) were used in the following experiments to determine the mechanism of action of TMBP on the promastigote forms.

The selectivity index (SI) of TMBP was expressed^45^ as:

$$SI=\frac{{CC}_{50}\,of\,TMBP\,on\,peritoneal\,macrophages}{{IC}_{50}\,of\,TMBP\,on\,promastigote\,forms}$$

#### Determination of mitochondrial membrane potential

Tetramethylrhodamine ethyl ester (TMRE) staining (Sigma-Aldrich, St. Louis, MO, USA) assessed the inner mitochondrial membrane potential. The promastigote forms (10^6^ cells/mL) were treated for 24 h with IC_50_ and 2 × IC_50_ of TMBP and then washed and incubated in 25 nM of TMRE for 30 min at 25 °C and analyzed in a fluorescence microplate reader (Victor X3, Perkin Elmer, Finland). *m*-Chlorophenylhydrazone carbonyl cyanide (CCCP; 100 μM) (Sigma-Aldrich) was used as a positive control to induce depolarization of the internal mitochondrial membranes. The readings were taken at an excitation wavelength of 480 nm and an emission wavelength of 580 nm^44^.

#### Production of reactive oxygen species (ROS) in promastigotes and *L. amazonensis*-infected macrophages

To evaluate the generation of ROS, the promastigote forms of *L. amazonensis* (10^6^ cells/mL) or *L. amazonensis*-infected macrophages (5 × 10^5^ cells/mL) were treated under the same conditions described in the anti-amastigote assay (see Sect. 2.1.10), and incubated at 25 °C for 24 h with TMBP at IC_50_ (0.86 μM) and 2 × IC_50_ (1.72 μM), or RMPI (Roswell Park Memorial Institute) 1640 medium. The parasites were then washed with PBS and loaded with 10 or 2 µM, respectively, of the diacetate permeate probe 2′,7′-dichlorofluorescein (H_2_DCFDA) (Sigma-Aldrich) diluted in DMSO (w/v) and incubated at 25 ºC in the dark for 30 min for the promastigote forms, and 45 min for the infected macrophages. ROS was measured by the increase in fluorescence caused by the conversion of the non-fluorescent H_2_DCFDA dye to the highly fluorescent 2,7-dichlorofluorescein (DCF), at an excitation wavelength of 488 nm and an emission wavelength of 530 nm, in a fluorescence microplate reader). Hydrogen peroxide (H_2_O_2_; 0.4%) was used as a positive control^46^.

#### Viability analysis of peritoneal macrophages

The evaluation of the cytotoxic effects of TMBP on peritoneal macrophages was carried out according to the assay procedure of Mosmann (1983)^47^, based on the mitochondrial oxidation of MTT (3-(4,5-dimethylthiazol-2-yl)-2,5-diphenyltetrazolium bromide) (Sigma-Aldrich). Macrophages (5 × 10^5^ cells/mL) were recovered from the peritoneal cavity of BALB/c mice with cold PBS supplemented with 3% of FBS and then cultured in 24-well multiplates with 200 μL of RPMI 1640 medium (w/v) (10% FBS) for 24 h (37 °C, 5% CO_2_). Adherent cells were incubated with TMBP (0.03; 0.06; 0.12; 0.24; 0.49; 0.98; 1.95; 3.90; 7.80; 15.6; 31.3; 62.5; 125 µM) and cultured for 24 h under the same conditions. After that, MTT (5 mg/mL) was added and left for 3 h. Untreated cells were used as control; DMSO 0.015% (v/v) as a vehicle and H_2_O_2_ 0.4% (v/v) as a positive control. The plates were read in a spectrophotometer (Thermo Scientific, Multiskan GO) at 550 nm.

#### Hemolytic assay

The hemolytic capacity on erythrocytes was measured to assess TMBP toxicity. Sheep blood (Ethics Committee for Animal Experimentation at Universidade Estadual de Londrina: no. 4496.2018.81) was collected in a heparinized vacuum tube and washed three times with PBS (123 g for 10 min). The treatments with TMBP (0.03; 0.06; 0.12; 0.24; 0.49; 0.98; 1.95; 3.90; 7.80; 15.6; 31.30; 62.50; 125 µM) were incubated 1:1 with a 2% erythrocyte suspension in a final volume of 200 µL at 37 °C for 3 h (5% CO_2_) in a 96-well multiplate. PBS was used as a control, DMSO 0.015% as a vehicle, and distilled water as a positive control for hemolysis. The plates were centrifuged (123 g for 10 min), and the supernatants collected and analyzed by reading the absorbance at 550 nm^46^.

#### Anti-amastigote assay

Peritoneal cells of BALB/c mice (5 × 10^5^ cells/mL) were cultured in 24-well plates as previously described by Gonçalves et al. (2018). After infection, non-internalized promastigotes were removed by washing with PBS, and the adherent cells treated with TMBP at IC_50_ (0.86 μM) and 2 × IC_50_ (1.72 μM) or RPMI 1640 medium (control) for 24 h (37 °C, 5% CO_2_). The cells were next stained with Giemsa (Laborclin, Pines-PR, Brazil), and 20 fields analyzed under oil immersion using an optical microscope (Olympus BX41, Olympus Optical Co., Ltd., Tokyo, Japan) to determine the percentage of infected macrophages, and the number of amastigotes per macrophage after 24 h of treatment. Untreated infected macrophages were used as a control, DMSO 0.015% as a vehicle, and 1 μM AmB as a positive control^44^.

### Statistical analysis

All data were expressed as the mean ± SEM. Data were analyzed using GraphPad Prism statistical software (GraphPad Software, Inc., USA, 500.288). Significant differences between the groups were determined through one-way ANOVA, followed by Tukey's test for multiple comparisons. Differences were considered statistically significant upon *p* < 0.05. At least three independent experiments were performed, each with duplicate datasets.

### Molecular modelling

#### Molecular docking procedures

The structural model of trypanothione reductase from *L. amazonensis* (LaTR) was built by homology modeling as described previously by Camargo et al.^48^ using trypanothione reductase from *Crithidia fasciculata* as a template crystal structure^49^ (PDB ID: 1TYP, Resolution: 2.80 Å). The structural model of Gp63 (leishmanolysin) from *L. amazonensis* (LaGp63) was built by homology modeling as described previously by Santiago-Silva et al.^50^ using Gp63 from *Leishmania major* as a template crystal structure^18^ (PDB: 1LML, resolution: 1.86 Å).

The ligand preparation and molecular docking were carried out by the GOLD v. 2020.1 (*Genetic Optimization for Ligand Docking*) program and four-functions scores were applied: GoldScore^51^, ChemScore^52^, ASP^53^, and ChemPLP^54^. We performed clustering analysis for LaTR^55^ and consensus docking for LaGp63^56^ to identify probable best-scored pose results. The ligands were subjected to 50 iterative runs.

The region of interest for LaTR was centered on the flavin ring of FAD (flavin adenine dinucleotide) at coordinates x: 60.247, y: 18.851, z: 7.602 within a 25 Å radius. The region of interest from Gp63 was centered on Zn^2+^ at x: 19.396, y: 43.866, z: 16.941 within a 15 Å radius. The best-scored pose for which docking results was considered by the root-mean-square-deviation (RMSD) calculation, was carried out using the Discovery Studio Visualizer (Dassault Systèmes BIOVIA, Discovery Studio Modeling Environment, Release 2017, San Diego: Dassault Systèmes, 2016), and the analysis of intermolecular interactions performed by the PyMOL program v. 2.5^57^.

## Conclusion

For the first time, this study has identified the promising anti-leishmania potential of the biphenyl TMBP against promastigote forms of *L. amazonensis* as it showed high activity (IC_50_ = 0.48 at 0.86 μM) in the shortest time of treatment, 24 h, displaying dose-dependent behavior. In addition, at the tested concentrations of TMBP, this biphenyl compound was 62.7 times more selective for promastigotes than the macrophage cells and did not cause hemolysis of sheep erythrocytes, indicating low cytotoxicity. TMBP-induced cell death in promastigotes was due to the generation of ROS, as well as mitochondrial dysfunction. TMBP was able to act on the intracellular parasite, reducing the number of viable amastigote forms within macrophages, suggesting that it can diffuse through the cell membrane, acting directly on the target, without causing considerable toxic effects on the host cell. Analysis of the consensual docking simulations on the LaTR and LaGp3 targets suggested that this biphenyl compound makes significant molecular interactions to inhibit these enzymes, with TMBP having a greater affinity for the LaGp63 target. The observed interactions occur nearby the Zn^2+^ ion in the case of LaGp3, while for LaTR, the interactions are nearby the cofactor, FAD. These results demonstrated the crucial participation of the hydroxyl group of TMBP, as most important in binding interactions between the ligand and both enzyme targets, indicating a possible pharmacophoric group of this compound.

## Data Availability

The authors can confirm that all relevant data are included in the article.
